# Engineering of a Bispecific Nanofitin with Immune Checkpoint Inhibitory Activity Conditioned by the Cross-Arm Binding to EGFR and PDL1

**DOI:** 10.3390/biom13040636

**Published:** 2023-03-31

**Authors:** Perrine Jacquot, Javier Muñoz-Garcia, Maurine Fleury, Denis Cochonneau, Rémi Gaussin, Elise Enouf, Caroline Roze, Emilie Ollivier, Mathieu Cinier, Dominique Heymann

**Affiliations:** 1 Affilogic SAS, 24 rue de la Rainière, 44300 Nantes, France; 2 Tumor Heterogeneity and Precision Medicine Laboratory, Institut de Cancérologie de l’Ouest, Université de Nantes, 44805 Saint-Herblain, France; 3 UMR 6286, US2B, CNRS, Nantes Université, 44322 Nantes, France

**Keywords:** epidermal growth factor receptor (EGFR), programmed cell death ligand 1 (PDL1), bispecific Nanofitin, tumor specific, immune checkpoint inhibitor (ICI)

## Abstract

Re-education of the tumor microenvironment with immune checkpoint inhibitors (ICI) has provided the most significant advancement in cancer management, with impressive efficacy and durable response reported. However, low response rates and a high frequency of immune-related adverse events (irAEs) remain associated with ICI therapies. The latter can be linked to their high affinity and avidity for their target that fosters on-target/off-tumor binding and subsequent breaking of immune self-tolerance in normal tissues. Many multispecific protein formats have been proposed to increase the tumor cell’s selectivity of ICI therapies. In this study, we explored the engineering of a bispecific Nanofitin by the fusion of an anti-epidermal growth factor receptor (EGFR) and anti-programmed cell death ligand 1 (PDL1) Nanofitin modules. While lowering the affinity of the Nanofitin modules for their respective target, the fusion enables the simultaneous engagement of EGFR and PDL1, which translates into a selective binding to tumor cells co-expressing EGFR and PDL1 only. We demonstrated that affinity-attenuated bispecific Nanofitin could elicit PDL1 blockade exclusively in an EGFR-directed manner. Overall, the data collected highlight the potential of this approach to enhance the selectivity and safety of PDL1 checkpoint inhibition.

## 1. Introduction

Although the treatment of solid tumors has recently progressed, with the development of various antibodies targeting tumor cells, the tumor microenvironment or mobilizing the host immune system still remains a challenge. Antigenic heterogeneity, parallel pro-tumorigenic signaling pathways and immunosuppressive tumor microenvironment are examples of drug resistance mechanisms that limit the efficacy of antibody-based monotherapies for the treatment of solid tumors [1]. Dose-limiting toxicity is another limiting factor for the efficacy of several immunotherapies. Simultaneous targeting of several tumor antigens using bi- or multispecific biologics has emerged as a promising strategy in cancer therapy for increasing potency as well as decreasing potential problems of drug resistance and adverse events related to off-tumor targeting [2]. While many bi- and multispecific formats vary in size, arrangement, valency, flexibility, and the geometry of their binding modules (derived from the antibody structure) [3,4], the high modularity of non-immunoglobulin alternative scaffolds make them attractive building blocks for the engineering of multispecific molecules [5,6,7,8,9,10].

Nanofitins are small (66 amino acids, 7 kDa), single-chain, hyperthermostable affinity proteins derived from Sac7d [11], a histone-like protein composed of a β-barrel capped on the back with a C-terminal α-helix and isolated from the hyperthermophilic archaeon *Sulfolobus Acidocaldarius*. Libraries of Nanofitins result from the randomization of the natural DNA-binding site of Sac7d, from which high-specificity binders to different molecules can be selected [12,13,14,15,16]. Nanofitins exhibit very high stability in a wide range of temperatures and pH [17,18]. They lack cysteine and post-translational modifications, which allow their soluble expression as much in eukaryotic as in prokaryotic systems. Owing to the location of their N- and C-termini ends on the opposite face of their variable domain, Nanofitins can be easily assembled into multispecific molecules using straightforward molecular approaches [13] while preserving the original pharmacologic and stability properties of the parent proteins. 

In a previous study, we demonstrated the additive anti-tumor effect of a bispecific Nanofitin, engineered by the genetic assembly of the two Nanofitin modules B11 and A-C2, respectively, enabling the neutralization of the immune checkpoint PDL1 and the polarization of tumour-associated macrophages to the anti-tumoral M1-like phenotype [13]. Due to their overall role in cancer immunity, modulation of the tumor-associated macrophages has been proposed as a strategy to improve the response rate to anti-PD1/PDL1 therapies [19]. Another means of improving anti-PD1/PDL1 therapies leans toward preventing their on-target/off-tumor binding and the subsequent occurrence of immune-related adverse events (irAEs). The latter could be achieved by promoting their tumor partitioning-dependent activation by tumor-associated antigen (TAA) targeting [20,21,22]. To this end, Koopmans et al. described the development of a bispecific antibody targeting both PDL1 and EGFR, hence driving the PDL1 blockade to EGFR-overexpressing cancer cells selectively [20]. EGFR has the advantage of being a well-established oncogenic TAA whose overexpression has been reported in numerous cancers [23,24,25,26,27,28], including some responsive to both anti-EGFR and anti-PD1/PDL1 therapies. We have previously described the development of a human and murine cross-reactive anti-EGFR Nanofitin named B10 and demonstrated its ability to target an EGFR-positive tumor in vivo [14] selectively. In this study, we explore the engineering of a bispecific Nanofitin bridging the previously described anti-EGFR B10 [14] and anti-PDL1 B11 [13] Nanofitin modules. We characterize the affinity of the bispecific Nanofitin for each target and its ability to target EGFR and PDL1-positive tumor cells selectively. Ultimately, we demonstrate the efficacy of the bispecific Nanofitin at promoting T cell anti-tumor cell cytotoxic activity in a TAA-conditioned manner. 

## 2. Materials and Methods

### 2.1. Plasmid Construct, Expression and Purification of Proteins

Monomeric Nanofitin constructs against EGFR and PDL1 were developed and named B10 and B11, respectively. Dimeric and monospecific Nanofitins were obtained by the fusion of B10 and B11 to the same irrelevant Nanofitin iNF (B10-iNF and iNF-B11). Dimeric and bispecific B10-B11 Nanofitin were also developed. All DNA constructs were obtained by gene synthesis (Genscript) with codon optimization for *E. coli* expression, fused at the N-terminal and C-terminal with the respective hexahistidine and hemagglutinin tags and cloned in the pET21a vector between the NdeI and HindIII restriction sites. The BL21 Gold (DE3) strains were transformed by heat shock and incubated overnight at 37 °C in 10 mL of 2YT, 1% glucose, 5 ng/mL Tetracycline and 100 ng/mL Ampicillin (growing medium) under gentle agitation. Then, the overnight pre-culture was diluted in 200 mL of growing medium and incubated at 37 °C under gentle agitation with the regular measurement of the OD at 600 nm. When the exponential phase was reached, the protein expression was induced by adding 0.5 mM of IPTG (Merck, Molsheim, France, #16758-10G) for 4 h at 30 °C. After chemical lysis for 15 min, the proteins were purified by IMAC with the NGC Quest 10 Plus System (HisTrapHP, Dutscher, Bernolsheim, France, #17-5248-02) and dimeric proteins were purified by size exclusion chromatography using a HiLoad 26/600 Superdex 75 pg. All proteins were formulated in PBS1X 0.1M L-Arginine, 40 mg/mL Trehalose and 0.01% Tween and filtered (Acrodisc^®^ membrane mustang^®^ E, #514-4235) to remove endotoxins below 10 EU/mg. 

### 2.2. Quality Control by Sodium Dodecyl Sulfate-Polyacrylamide Gel Electrophoresis

Two µg of each protein were mixed with a lane marker non-reducing sample buffer (Thermo Fisher Scientific, Villebon-sur-yvete, France, #39001) and were denatured by heating for 5 min at 95 °C. Samples and the PageRuler Plus Prestained Protein Ladder (Thermo Fisher Scientific, #26619) were loaded on Mini-protean TGX pre-casted gels 8–16% (Bio-Rad, Marnes-La-Coquette, France, #4561106) and the migration was carried out for 25 min in a Tris/Glycine/SDS running buffer (VWR, Rosny-sous-Bois, France, #J61006K3) with a constant voltage of 200 volts. Finally, protein bands were stained with ReadyBlue Protein gel stain (Merck, #RSB-1L) for one hour and then washed overnight with water.

### 2.3. Biolayer Interferometry Analyses 

Nanofitin affinity and targeting capacity were determined by biolayer interferometry (BLI) on an Octet Red instrument (Fortebio). All biolayer interferometry analyses were performed in 96 multi-well plates (Dutsher, #655900) at 30 °C with a continuous shake speed. The binding kinetic parameters of anti-EGFR Nanofitins were determined by loading of recombinant human EGFR Fc chimera protein (R&D, Rennes, France, #344-ER-050) (10 µg/mL) at 2 nm on protein A biosensors (Sartorius, Dourdan, France, #18-5012). Similarly, the binding kinetic parameters of anti-PDL1 Nanofitins were determined by the loading of recombinant human PDL1/B7-H1 Fc chimera protein (R&D, #156-B7-100) (5 µg/mL) at 2 nm on protein A biosensors. All measured steps were performed in TBS1X containing 0.002% Tween 20 and 0.01% BSA. Between each measurement, biosensors were regenerated using three cycles of alternating washes for 10 s in Glycine 10 mM pH 2 and in TBS1X. The biosensor unexposed to Nanofitin was used as a background reference. Sensorgrams were obtained after a reference subtraction, a background correction, a smoothing with the Savitzky–Golay algorithm and a fitting with a 1:1 model using the Octet Data Analysis software 7.1.

To determine the Nanofitin capacity to simultaneously target PDL1 and EGFR, recombinant human EGFR Fc chimera protein was diluted to 10 µg/mL and loaded onto a protein A biosensor at 2 nm. After the biosensor’s equilibration for 5 min, two association steps were performed; the first was the biosensor’s exposure to 10 µg/mL of Nanofitin, and the second was the biosensor’s exposure to a mix of 10 µg/mL of Nanofitin and 10 nM of recombinant human PDL1/B7-H1 His-tag protein (R&D, #9049-B7-100). The dissociation step was measured for 5 min. All steps were performed in TBS1X containing 0.002% Tween 20 and 0.01% BSA. Additional details for the biolayer interferometry analyses are reported in supporting materials and methods.

### 2.4. Enzyme-Linked Immunosorbent Assays

Biochemical characterization of Nanofitins was performed by measuring their IC50 for PDL1. Ninety-six multi-well plates were coated for 1 h with recombinant human PD-1 His-tag protein (R&D, #8986-PD-100) diluted at 1 µg/mL in TBS1X buffer. After washing with TBS1X buffer, the non-specific binding sites were blocked with TBS1X buffer containing 0.5% Bovine Serum Albumin (BSA) for 1 h. Serial dilutions of the Nanofitins were then pre-incubated with 10nM of biotinylated recombinant human PDL1/B7-H1 Fc chimera protein (R&D, #156-B7-100) in 96 multi-well low-binding plates (VWR, #269620). The plates were washed with TBS1X buffer containing 0.1% Tween and incubated with the mix of PDL1 and Nanofitins dilutions for 1 h before incubation with HRP-Streptavidine antibody (Abcam, Amsterdam, Netherlands, #ab100548) for 1 h. The reaction was started by adding TMB substrates and stopped by adding HCl. All incubation steps were carried out at room temperature, and all plates were read at 450 nm (Varioskan system, Thermo Fisher Scientific, #3001-2017). Additional details for the enzyme-linked immunosorbent assays are reported in supporting materials and methods.

### 2.5. Cell Culture

All cell lines were obtained from American Type Culture Collection (ATCC) and were cultured in a 37 °C incubator with 5% CO_2_ saturated in humidity. Human A431 (#CRL-1555, epidermoid carcinoma), MNNG-HOS (#CRL-1543, osteosarcoma) and U2OS (#HTB-96, osteosarcoma) cells were cultured in DMEM with 5% FBS and 1% l-glutamine. Human Jurkat (#TIB-152, T lymphoblast) cells were cultured in RPMI with 5% FBS and 1% l-glutamine. Human MDA-MB-231 (#HTB-26, breast adenocarcinoma) cell line was cultured in l-15 medium with 5% FBS and 1% l-glutamine. All cell lines were tested negative for mycoplasma before use.

### 2.6. Cell-Surface Binding by Flow Cytometry

Proliferating cells were washed with PBS and detached by Versene. After a 5 min centrifugation at 450× *g*, the cell pellets were washed twice with cold PBS and then resuspended at 2 × 10^6^ cells/mL. Cells were distributed in 96 multi-well plates and incubated for 15 min in PBS1X-1%BSA. The expression level of EGFR and PDL1 on cells were studied by respectively incubating cells with 100 µL of recombinant Alexa Fluor 488 anti-PD-L1 antibody (Abcam, #ab209959, 1/50 dilution) and PE anti-human EGFR antibody (Biolegend, San Diego, CA, USA, #352903, 1/20 dilution). In order to evaluate the PDL1 and EGFR level expression on cells, the respective control antibodies were used: recombinant Alexa Fluor 488 Rabbit IgG, monoclonal [EPR25A] (Abcam, #ab199091), and PE mouse IgG1 kappa isotype control antibody (Biolegend, #400111). The cell binding capacity of Nanofitin was analyzed by incubating cells with 100 µL of Nanofitin at 10 µM followed by the addition of DyLight650 anti-HA tag antibody (Abcam, #ab117515, 1/200 dilution). In order to evaluate the Nanofitin binding capacity to cells, the control condition was performed by adding the DyLight650 anti-HA tag antibody on cells without previous incubation of Nanofitin. The FL-1 (Alexa Fluor 488), FL-2 (PE) and FL-4 (DyLight 650) fluorescence on cells were analyzed using flow cytometry (BD Accuri^TM^ C6 Plus System). Additional information about cell-surface binding assays by flow cytometry are reported in supporting materials and methods.

### 2.7. Western Blot Analysis

Proliferating A431 cells were seeded in 6-well plates and cultured in DMEM, 10% FBS, and 1% l-glutamine medium for 24 h in a 37 °C incubator with 5% CO_2_ saturated in humidity. After overnight culture in DMEM, 1% FBS, 1% l-glutamine medium, A431 cells were incubated in DMEM, 0% FBS, 1% l-glutamine medium with 100 µg/mL or in the absence of Cetuximab antibody or anti-EGFR Nanofitin for 4 h. Then 10 ng/mL of EGF (Creative Biomart, Shirley, USA, #EGF-04H) was added for 15 min. Cell lysis was performed at 4 °C for 1 h in RIPA buffer supplemented with phosphatase (Thermo Fisher Scientific, #78420, 1/1000 dilution) and protease (Merck, #S8820) inhibitors. The extracted proteins were diluted with loading buffer and denatured at 95 °C for 5 min. After migration on a 12% SDS-PAGE gel, transfer to PVDF membrane was performed at 4 °C. Blocking was performed in 5% BSA TBS-Tween buffer for 1 h. Overnight incubation with the anti-EGFR antibody at 4 °C (Cell Signaling, Ozyme, Saint-Cyr-L’Ecole, France, #2232S, 1/1000 dilution) was followed by incubation of HRP-linked anti-rabbit IgG antibody (Cell Signaling, #7074S, 1/1000 dilution) for 1 h. Revelation of phosphorylated EGFR protein was performed after the membrane dehybridization by overnight incubation with the Phospho-EGF Receptor (Tyr845) antibody (Cell Signaling, #2231S, 1/1000 dilution) followed by 1 h incubation with HRP anti-rabbit IgG antibody (Cell Signaling, #7074S, 1/1000 dilution). Revelations were performed using ChemiDocTM MP (Bio-Rad). 

### 2.8. PD1/PDL1 Blockade Bioassay

The blockade of PD-1/PD-L1 interaction was assessed using a commercially available PD-1/PD-L1 blockade bioassay (Promega). PDL1 aAPC/CHO-K1 were cultured for 20 h and then co-cultured with Jurkat/PD1/NFAT/luc T cells for 6 h and with Nanofitin constructs. The co-culture of both cell lines inhibits TCR signaling and NFAT-mediated luciferase activity of Jurkat/PD1/NFAT/luc T cells. The inhibition of PD1/PDL1 interaction by the Nanofitins results in the activation of TCR signaling and the release of luciferase. 

### 2.9. Agilent X-Celligence Real-Time Cell Analysis (RTCA)

In order to determine the Nanofitins’ impact on tumor cell proliferation in the presence of immune cells, 5 × 10^3^ MNNG-HOS cells were seeded in 96 multi-well electronic plates (Agilent Technologies, Les Ulis, France, #05232376001). After 24 h, the media was removed and replaced with fresh media containing Nanofitins (10 µM). After 1 h of incubation, Jurkat cells were mixed with an anti-CD3 scFv (produced by Sinobiological). The MNNG-HOS cells were co-cultured with Jurkat cells at an E:T ratio of 10:1. The anti-CD3 scFv was used at a suboptimal concentration (75 ng/mL) in order to develop the background activity of Jurkat cells. The real-time kinetic proliferation of tumor cells was analyzed for 100 h with the RTCA system.

## 3. Results

### 3.1. The Bispecific Nanofitin B10-B11 Can Engage Simultaneously EGFR and PDL1

**Figure 1:**
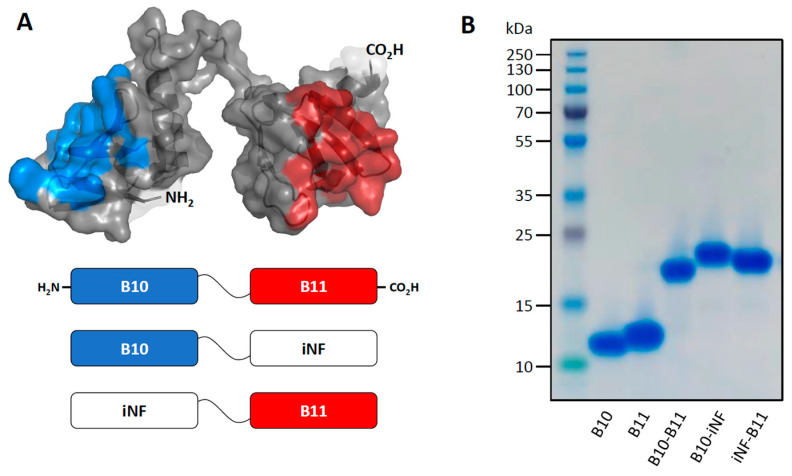
Representation and purity of the bispecific Nanofitin B10-B11 and relative control constructs. (**A**) Schematic representation of the topology of the dimeric Nanofitin constructions involved in the study and 3D model of the B10-B11 bispecific Nanofitin performed using MODELLER. The structure 1AZP was used as the initial scaffold to model by homology the Nanofitins B10 and B11. The paratope of B10 has been labelled in blue and that of B11 in red. (**B**) SDS-PAGE analysis of the Nanofitin constructs B10, B11, B10-B11, B10-iNF and iNF-B11.

The generation of the anti-EGFR and anti-PDL1 Nanofitin, respectively named B10 and B11, has been previously described [17,18]. In the present work, we investigated the generation of a tumor-selective bispecific molecule by the genetic assembly of these two Nanofitins (Figure 1A). Unless otherwise specified, the two Nanofitin modules in the dimeric constructions were separated with a 15 mers peptide linker. A Nanofitin that targets the GFP [18] (iNF) was also involved in the study to engineer the dimeric control constructs iNF-B11 and B10-iNF (Figure 1A). All the different Nanofitin products were obtained at high purity as demonstrated by SDS-PAGE (Figure 1B), and their binding profiles on either or both EGFR and PDL1 were assessed by biolayer interferometry.

**Figure 2:**
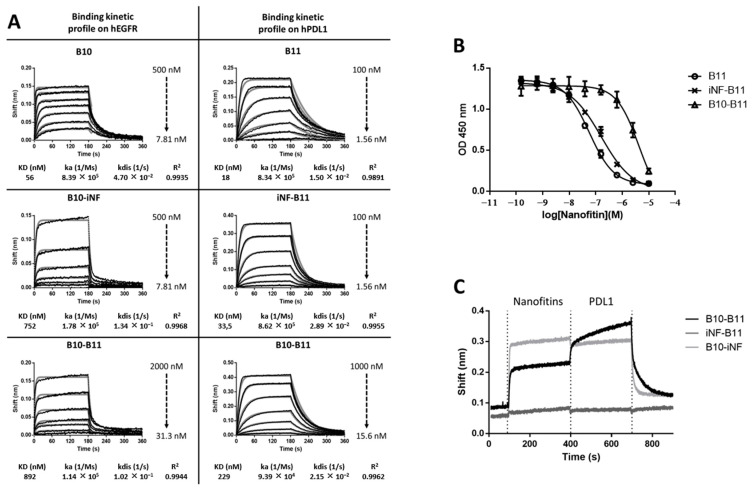
Biochemical analysis of monomeric and dimeric anti-EGFR and anti-PDL1 Nanofitins. (**A**) Biolayer interferometry sensorgrams and binding kinetic parameters of B10, B10–iNF and B10–B11 on EGFR as well as B11, iNF-B11 and B10–B11 on PDL1. (**B**) Evaluation of the B11-based constructs at neutralizing PD1/PDL1 interaction in a competitive ELISA assay. (**C**) Biolayer interferometry sensorgrams showing the co-engagement of both EGFR and PDL1 by the B10–B11 bispecific Nanofitin. Fittings (1:1 model) are represented as solid gray lines.

Each Nanofitin module appeared to be selective for its target, with binding on EGFR or PDL1 observed only in the presence of B10 or B11, respectively (Figure S1). B10 and B11 exhibit an affinity for their respective targets of 56 nM and 18 nM (Figure 2A). When fused at its C-terminal end with another Nanofitin, B10 displayed a lower affinity for EGFR, as shown with the constructions B10–iNF (752 nM) and B10–B11 (892 nM). Comparison of the dose–response curves of the anti-EGFR-based constructs in ELISA further confirmed the lower affinity of the dimeric constructs for EGFR (Figure S2). Interestingly, the affinity of B11 for PDL1 appeared to be differentially impacted by the fusions at its N-terminal end. The construct iNF-B11 showed an affinity for PDL1 (33.5 nM) comparable to that of B11, while B10–B11 exhibited a lower affinity of 229 nM. The potency of the B11-based constructs at neutralizing PD1/PDL1 interaction in a competitive ELISA assay was found to be correlated with the ranking of their affinities for PDL1 (Figure 2B). Ultimately, we demonstrated by biolayer interferometry that B10 and B11 were able to engage their targets when fused to each other simultaneously. B10–B11 could be captured on EGFR-loaded biolayer sensors, enabling the newly functionalized biosensors to capture PDL1. Alternatively, the substitution of the anti-EGFR B10 and anti-PDL1 B11 Nanofitins with the control Nanofitin iNF prevented either the initial capture on EGFR-loaded biosensors (iNF-B11) or the later capture of PDL1 (B10-iNF) (Figure 2C).

### 3.2. Cell Binding of the Bispecific Nanofitin B10–B11 Is Conditioned by the Cross-Arm Binding of EGFR and PDL1

**Figure 3:**
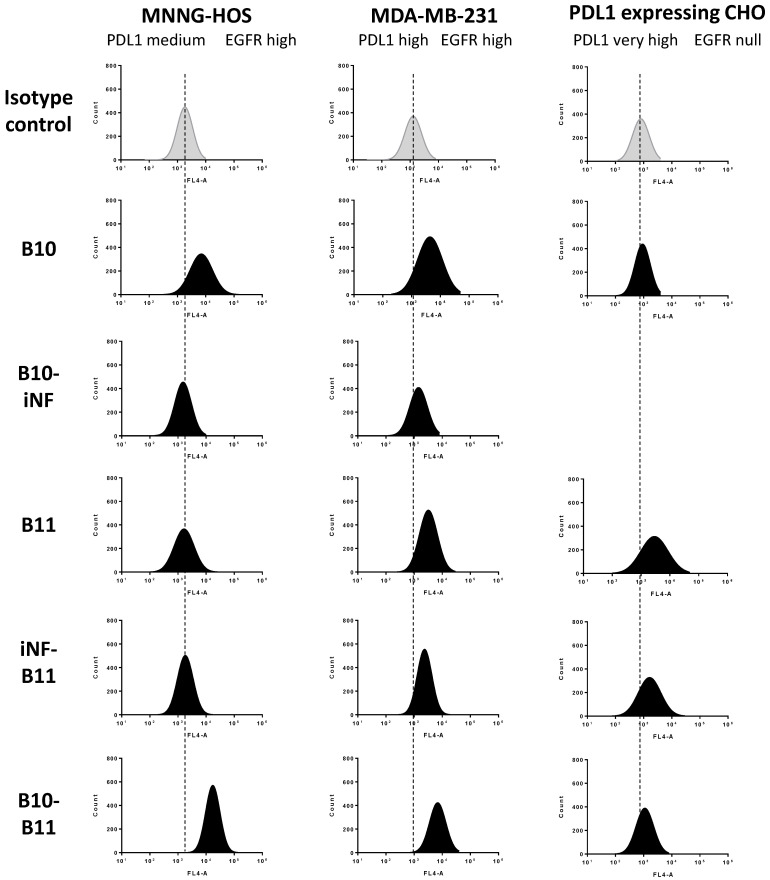
Cell labelling efficiency evaluation by flow cytometry of B10, B11, iNF-B11, B10-iNF and B10–B11 on the EGFR and PDL1 positive tumor cell lines MDA-MB-231 and MNNG-HOS as well as on the PDL1^+^/CHO cell line engineered to overexpress PDL1 stably. In grey: isotype control; In black: the Nanofitin and secondary antibody; Dotted lines: alignment to isotype control.

The tumor cell labelling efficiency of B10, B11, iNF-B11, B10-iNF and the bispecific construct B10–B11 was evaluated on the EGFR and PDL1 positive tumor cell lines MDA-MB-231 and MNNG-HOS, as well as on the PDL1^+^/CHO cell line engineered to overexpress PDL1 stably (Figure 3). The expression profile for EGFR and PDL1 of these cell lines was confirmed by flow cytometry (Figures S3 and S4). The MDA-MB-231 and MNNG-HOS cell lines were found to both display a high level of EGFR expression, while the PDL1^+^/CHO cell line was negative to EGFR expression. The PDL1 expression level of the MNNG-HOS, MDA-MB-231 and PDL1^+^/CHO cell lines was ranked from medium to very high in that order.

The labelling efficiency of the monomeric Nanofitin B10 and B11 on these three cell lines followed their EGFR and PDL1 expression profiles. The B10 Nanofitin provided a high labelling of the MNNG-HOS and MDA-MB-231 cells, but did not bind to the EGFR-negative PDL1^+^/CHO cells. B11 was able to engage both the high and very highly PDL1 expressed cell lines MDA-MB-231 and PDL1^+^/CHO, but not the MNNG-HOS, showing a medium PDL1 expression level. 

The affinity of B10–B11 for EGFR and PDL1 is lower than their respective monomers (Figure 2A). Despite a high expression of EGFR, B10-iNF was inefficient at labelling the MNNG-HOS and MDA-MB-231 cells, which could be attributed to its lower affinity for EGFR compared to B10. As a consequence, its binding on the PDL1^+^/CHO cells was not evaluated. The similar affinity reduction of B10–B11 for EGFR could suggest the same monovalent binding capacity of this Nanofitin dimer. The iNF-B11 showed a similar affinity for PDL1 as B11 and shared a similar cell binding profile as well, although with a slightly lower labelling efficiency than B11 on both the MDA-MB-231 and PDL1^+^/CHO cells. Conversely, B10-B11, with a lower affinity for PDL1, was found to be poorly efficient at engaging the PDL1/CHO cells despite PDL1 overexpression. Taken together, these results support the fact that the affinity for EGFR and PDL1 of the B10–B11 bispecific Nanofitin does not support the monovalent engagement of cells overexpressing a high level of either receptor. 

Interestingly, a high cell-labelling index was observed with B10-B11 on both MDA-MB-231 (EGFR/PDL1, high/high) and MNNG-HOS (EGFR/PDL1, high/medium) cell lines, suggesting a binding capacity mediated by the simultaneous engagement of the two receptors highly expressed on these target cells. Conversely, this same trend was not observed on U2OS (EGFR/PDL1 low/high), which suggests that a threshold EGFR expression is required to allow efficient cell engagement and support the inability of the construct to engage healthy cells (Figure S5). Additionally, this suggests that the simultaneous engagement of the bispecific Nanofitin relies on the high expression of both EGFR and PDL1 on cells.

### 3.3. Decreasing the Linker Size Shows Opposing Effect on Affinity and Cell Binding Efficiency

**Figure 4:**
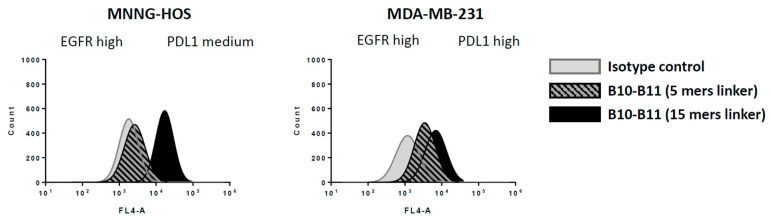
Comparison of the cell labelling efficiency of the bispecific Nanofitin B10-B11 when engineered with a 5 or 15 mers linker on MNNG-HOS and MDA-MB-231 cell lines.

We also investigated the influence of decreasing the spacer length (15 vs. 5 mers) between the two Nanofitin modules in the bispecific construction B10-B11 for its ability to engage cells expressing different levels of EGFR and PDL1. Affinities for EGFR and PDL1 of the cognate bispecific Nanofitin made with a 5 mers linker were also assessed by biolayer interferometry and evaluated at 245 and 34.6 nM, respectively (Figure S6). Notwithstanding its higher affinity for the targets, the 5 mers bispecific Nanofitin showed a lower binding on both MDA-MB-231 and MNNG-HOS cells than the 15 mers bispecific construct (Figure 4).

### 3.4. The Bispecific Nanofitin B10-B11 Displays Immune Checkpoint Inhibitory Activity in an EGFR-Dependent Manner

**Figure 5:**
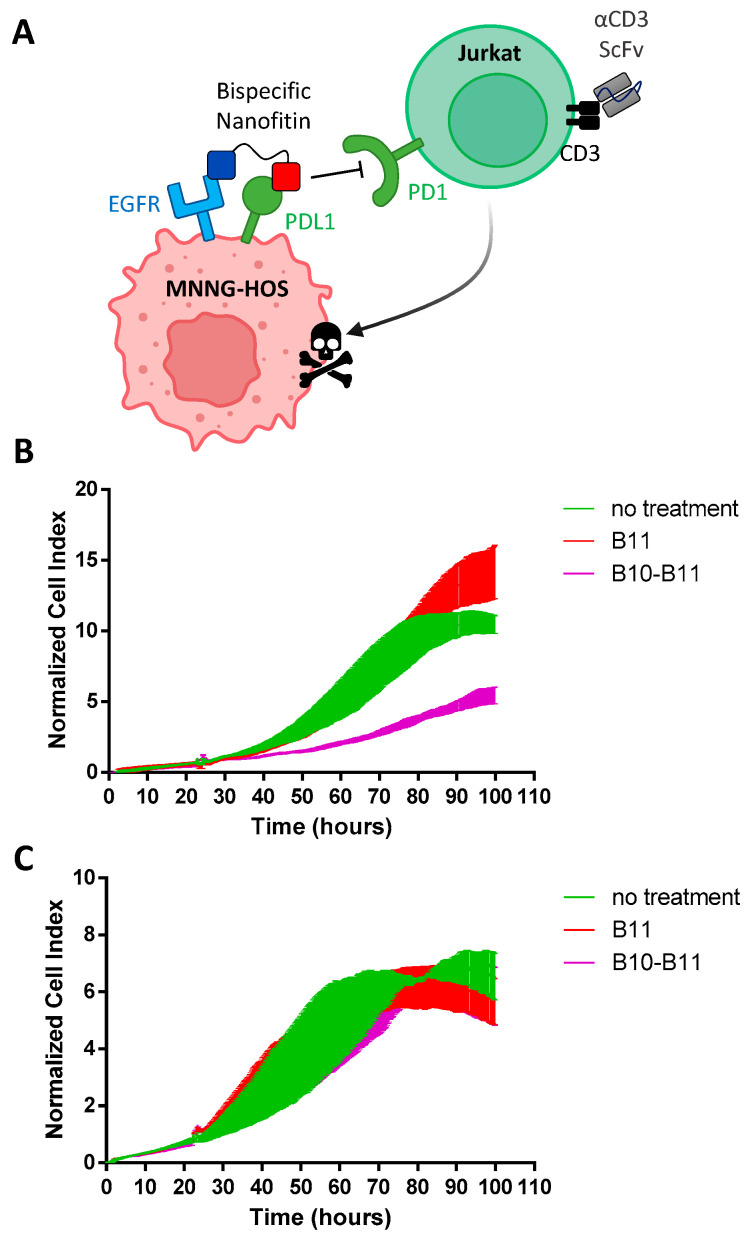
Real-time evaluation by impedancemetry of the effect of B11 and B10-B11 on MNNG-HOS tumor cells proliferation. (**A**) Schematic representation of the immune checkpoint inhibitory activity of B10-B11 leading to MNNG-HOS cell killing by co-cultured Jurkat cells activated by an anti-CD3 ScFv. (**B**) The real-time proliferation of MNNG-HOS cells co-cultured with Jurkat cells at an E:T ratio of 10:1. (**C**) The real-time proliferation of MNNG-HOS cells. In green: MNNG-HOS cells alone or co-cultured with Jurkat cells. In red: incubation in the presence of B11 (10 µM). In magenta: incubation in the presence of B10-B11 (10 µM).

**Figure 6:**
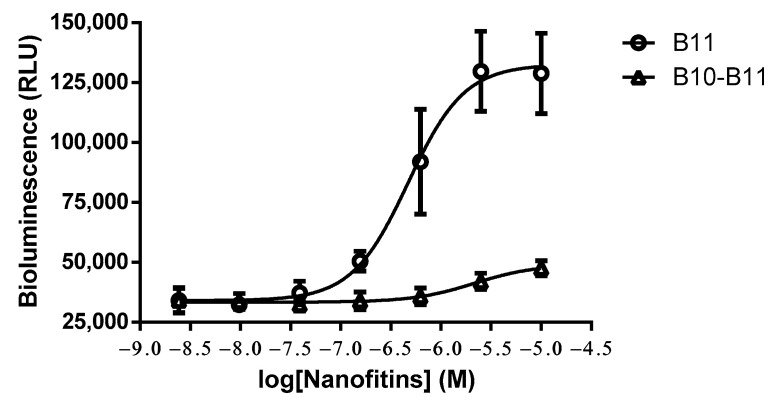
Monitoring of the immune checkpoint inhibition activity of B11 and B10-B11 using a PD1/PDL1 blockage bioassay (Promega).

The anti-tumor potential of the 15 mers B10-B11 bispecific Nanofitin was evaluated by impedancemetry by following over 100 min of the real-time proliferation of a co-culture with a 1:10 ratio of MNNG-HOS and activated Jurkat cells with an anti-CD3 ScFv (Figure 5A). Compared to the untreated co-culture, cell proliferation appeared to be significantly slowed down with incubation of the B10-B11 bispecific Nanofitin (10 µM) but not with the monospecific Nanofitin B11 (Figure 5B). We also demonstrated the B10-B11 bispecific Nanofitin’s anti-tumor activity dependence on the PDL1/PD1 inhibition of interaction. Indeed, neither B10-B11 nor B11 altered the proliferation of MNNG-HOS cells in the absence of Jurkat cells (Figure 5C). Moreover, for highly EGFR-expressing A431 cells, Western blot analysis illustrated the incapacity of the B10 Nanofitin to modify the EGFR phosphorylation mediated by EGF (Figure S7). Alternatively, we used the PD1/PDL1 blockade bioassay from Promega to assess the immune checkpoint inhibitory activities of B11 and B10-B11 in a co-culture between the engineered CHO cell line overexpressing PDL1 and engineered reporter Jurkat cells. PD-1/PD-L1 interaction inhibits TCR signaling and NFAT-mediated luciferase activity in the Jurkat cells. In this assay, B11 displayed an IC50 of about 472 nM, while the activity of B10-B11 was barely detectable, even at the highest concentration investigated (10 µM) (Figure 6). Overall, these results support the ability of the B10-B11 bispecific Nanofitin to neutralize PDL1 and promote T cell-mediated tumor cell death in a TAA-dependent manner. 

## 4. Discussion

Cancer development initially results from the failure of the immune system to eradicate the bad cells efficiently. This involves the deployment of resistance mechanisms [1] that can include the mounting of an immunosuppressive (T-cell exhaustion), immunotolerant (Treg upregulation) or immunodepleted (immune desert) tumor microenvironment. Re-education of the tumor microenvironment with immune checkpoint inhibitors (ICI) has demonstrated impressive efficacy, but with a relatively low response rate [29] and a high frequency of irAEs [30]. While combination immunotherapy targeting two different immune checkpoints can provide a significantly higher response rate, it also increases the occurrence of toxicity issues. In a clinical study evaluating the combination of Ipilimumab and Nivolumab, half of the patients experienced severe irAEs [31]. Better management of the toxicity profile of ICIs would facilitate their use in combination therapies. Currently approved ICIs are monoclonal antibodies. The occurrence of irAEs can be linked to their high affinity and avidity for their targets, fostering on-target/off-tumor binding and subsequent breaking of immune self-tolerance in normal tissues. Many different multispecific formats have been proposed to increase the tumor-cell selectivity of ICI therapies, notably by the use of asymmetric monovalent bispecific formats. These formats are deprived of avidity for a single molecular target, and their functional target engagement can be conditioned to the cross-arm binding of two heterologous targets, allowing then for a more selective biology [32,33]. This approach has been exploited for the development of the bispecific antibody MEDI5752 enabling PD1 blockade and the preferential neutralization of CTLA4 on tumor-infiltrated PD1^+^ antigen-experienced T-cells, compared to PD1^-^ T-cells [34]. From a different perspective, Koopmans et al. reported the engineering of a bispecific antibody targeting EGFR and PDL1 to direct PDL1 blockade to EGFR co-expressing tumor cells [20]. The bispecific antibody was constructed according to the taFv–Fc format that provides a symmetric tetravalent molecule by the fusion of two scFvs to an Fc fragment. Notwithstanding the clear demonstration of the selective anti-tumor activity of their bispecific molecule, it is unfortunate that neither the affinity of the different binding modules nor how the design prevented avidity was described. 

In this study, we investigated the generation of a bispecific molecule that includes the fusion of the two previously described anti-EGFR (B10) and PDL1 (B11) Nanofitin-based targeting modules [13,14], separated with a 15 mers linker. We demonstrated by biolayer interferometry that the fusion alleviated the binding affinity of the two Nanofitin modules and provided the bispecific molecule with the ability to engage the two targets simultaneously. In cell binding assays, the alteration of affinity of each binding module (892 nM for EGFR and 229 nM for PDL1) fully abrogated their monovalent engagement of cell surface EGFR or PDL1, even in a context of an elevated overexpression (B10-iNF for MNNG-HOS and MDA-MB-231 cell lines and B10-B11 for PDL1^+^/CHO cell line). On the contrary, the bispecific Nanofitin strongly labelled the two EGFR^+^/PDL1^+^ cell lines MNNG-HOS and MDA-MB-231, suggesting a specific cell engagement mediated by the cross-arm binding of the two molecular targets. Mazor et al. already described the increase in tumor cell selectivity via the simultaneous binding of two TAAs with a DuetMab format involving affinity-attenuated binding modules [32,35]. Harms et al. described a simulation study suggesting that efficient cross-arm binding can drive a hundredfold or greater improvement in inhibition of one TAA in the case of a higher expression (>10 fold) of the second TAA [36]. They also highlighted that modest affinity (10–100 nM) toward the TAA showing the highest expression level (10^5^–10^6^ TAA/cell) is sufficient to achieve maximum inhibition of the other TAA, with 100 nM being the lowest affinity they explored. The reported EGFR expression level of the MDA-MB-231 (5.2 × 10^5^ EGFR/cell [37]) falls within this range, and in our experiments, MNNG-HOS cells were found to have a similar, yet slightly higher, EGFR expression level. In the present study, the mechanism of action of the bispecific Nanofitin appeared mediated mainly by the inhibition of T cell anergy through the neutralization of PDL1 via the B11 Nanofitin in a dependent of a cross-arm binding with EGFR. Indeed, the inability of the anti-EGFR B10 Nanofitin to abolish the EGFR phosphorylation, similar to Cetuximab pre-treatment, suggests that this Nanofitin engages but does not alter the biology of EGFR. Zhou et al. discussed the impact of valency and affinity of proteins associated with EGFR signaling inhibition [38]. B10 Nanofitin is a monovalent protein with an affinity of 56 nM, about 3.6 times higher than the P2/4 scFv described by these authors that was insufficient to inhibit EGFR signaling as a monovalent protein but which demonstrated its high activity as a bivalent IgG. Moreover, because the B11 anti-PDL1 Nanofitin did not demonstrate anti-proliferative activity on HOS cells as a monomer and did not show neutralization potential as a bispecific protein on EGFR negative cells, we can suggest that pre-anchoring of Nanofitin on EGFR increases the neutralization potency of the PDL1 Nanofitin targeting module. We also demonstrated the inability of the bispecific Nanofitin to use the pre-anchoring activity in low EGFR expressing cells despite the high levels of PDL1, which support its selectivity for cancer cells with moderate to high levels of EGFR et PDL1 and a lack of engagement of healthy cells. It would be interesting to evaluate how increasing the affinity of the anti-EGFR or the anti-PDL1 Nanofitin modules in the bispecific construction can affect cell binding selectivity, as well as PDL1 blockade potency and anti-EGFR downstream signaling activity. As far as we are aware, simulation studies evaluating the impact of cross-arm binding were performed assuming an IgG structure, hence considering a fixed arm-to-arm distance of 125 Å [39]. In our case, the linker length can be adapted. When we compared our initial Nanofitin bispecific constructs with a similar one that differs only by the linker length (5 vs. 15 mers), we observed an inverse correlation between affinity and cell binding efficiency. Assuming a contour length of ~4 Å/amino acid [40], a fully stretched 5 mers linker will provide a spacing of the two Nanofitin modules of ~20 Å and the 15 mers of ~60 Å. The fusion with a shorter linker resulted in a lesser reduction of affinity of the Nanofitin modules (245 nM for EGFR and 34.6 nM for PDL1), which unexpectedly translated into a lesser cell binding efficiency on the two EGFR^+^/PDL1^+^ cell lines. This result suggested that a minimal linker length within the bispecific Nanofitin is required to allow efficient cross-arm binding of the two molecular targets on MNNG-HOS and MDA-MB-231, and it would be interesting to investigate whether this remains true with other cell lines exhibiting different expression levels of the two targets.

In conclusion, we investigated the generation of a bispecific Nanofitin by the simple and straightforward genetic fusion of two previously described anti-EGFR and anti-PDL1 Nanofitin modules. The attenuation of the binding affinity of the Nanofitin modules fully abrogated their monovalent binding on their respective target, conditioning the cell binding ability of the bispecific construct to tumor cells overexpressing the two targets. Additionally, we demonstrated that the bispecific Nanofitin could elicit a PDL1 blockade in an EGFR-directed manner. While the efficacy of the molecule remains to be demonstrated in vivo, the selectivity data collected highlight the potential of this approach to enhance the selectivity and safety of a PDL1 checkpoint blockade. 

## Data Availability

Not applicable.

## Supplementary Materials

The following supporting information can be downloaded at: https://www.mdpi.com/article/10.3390/biom13040636/s1; Figure S1: Evaluation of the binding specificity on EGFR and PDL1 of B10, B10-iNF, B11 and iNF-B11; Figure S2: ELISA dose-response curve on EGFR of the Nanofitin constructs B10, B10-iNF and B10-B11; Figure S3: Expression level of EGFR studied by flow cytometry on (A) MNNG-HOS, (B) MDA-MB-231 and (C) on PDL1 expressing CHO cell lines. In grey: isotype control; in black: anti-EGFR antibody; Figure S4: Expression level of PDL1 studied by flow cytometry on (A) MNNG-HOS, (B) MDA-MB-231 and (C) on PDL1 expressing CHO cell lines. In grey: isotype control; in black: anti-PDL1 antibody; Figure S5: Flow cytometry labelling efficiency evaluation of Nanofitins on U2OS non target cell line (Low EGFR, High PDL1). (A) Expression level of EGFR and PDL1. In grey: isotype control; In black: anti-EGFR or PDL1 antibody. (B) Cell labelling efficiency evaluation of B10, B11 and B10-B11 Nanofitins (10 µM). In grey: isotype control; In black: the Nanofitin and secondary antibody. Figure S6: Affinity of the bispecific Nanofitin B10-B11 (5 mers linker) for (A) EGFR and (B) PDL1. Figure S7: EGFR phosphorylation level in the presence or the absence of EGF, Cetuximab and B10 Nanofitin studied by Western Blot on A431 cell line.
