# Supplementary material for: Engineering of a Bispecific Nanofitin with Immune Checkpoint Inhibitory Activity Conditioned by the Cross-Arm Binding to EGFR and PDL1
Source: Biomolecules. 2023 Mar 31;13(4):636. doi: 10.3390/biom13040636 (PMC10135760; doi:10.3390/biom13040636)
Supplement: Supplementary file 1 [file biomolecules-13-00636-s001.zip › Supporting figures.pdf]

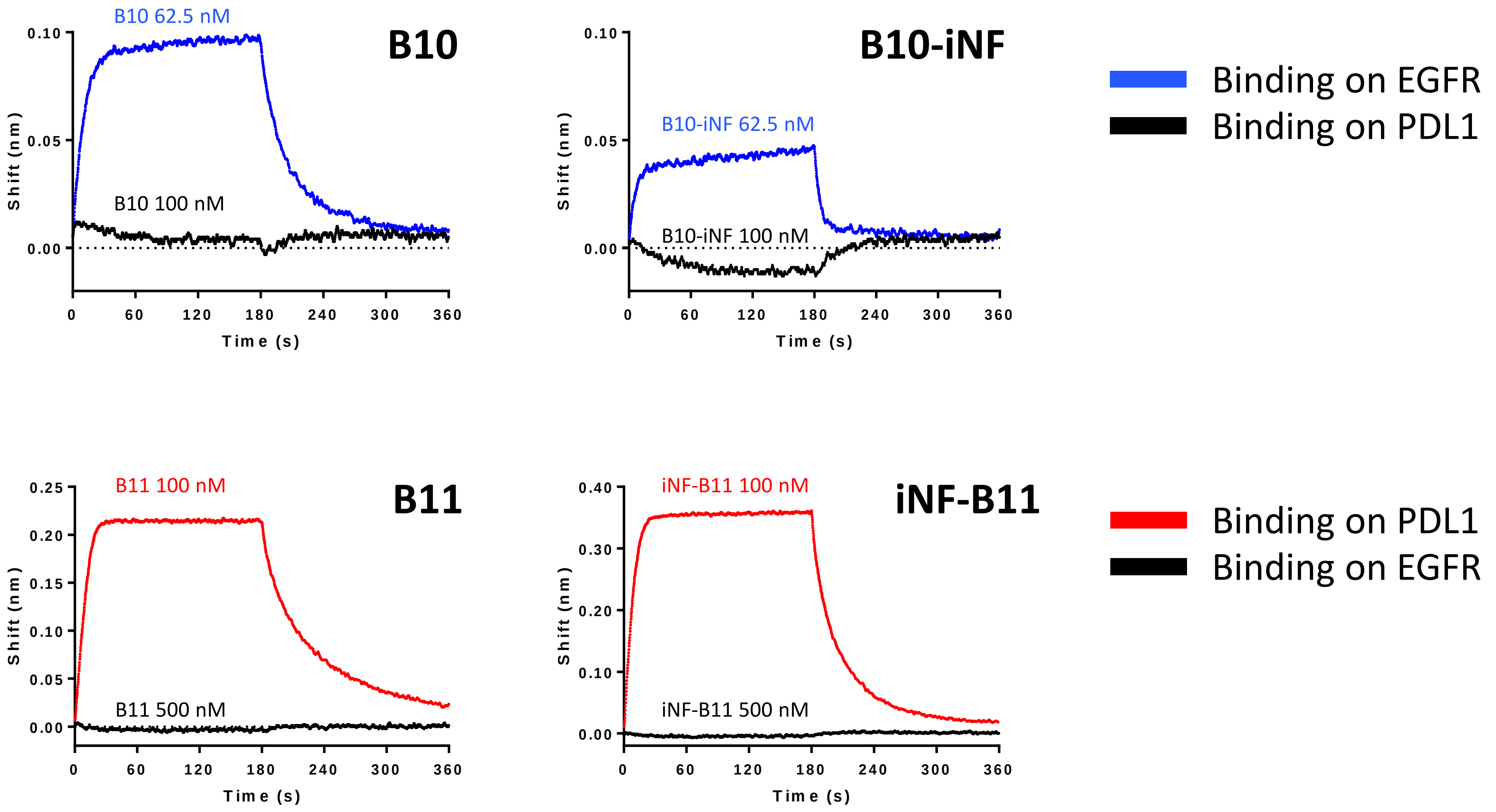


**<u>Supplementary figure S1:</u>** Evaluation of the binding specificity on EGFR and PDL1 of B10, B10-iNF, B11 and iNF-B11

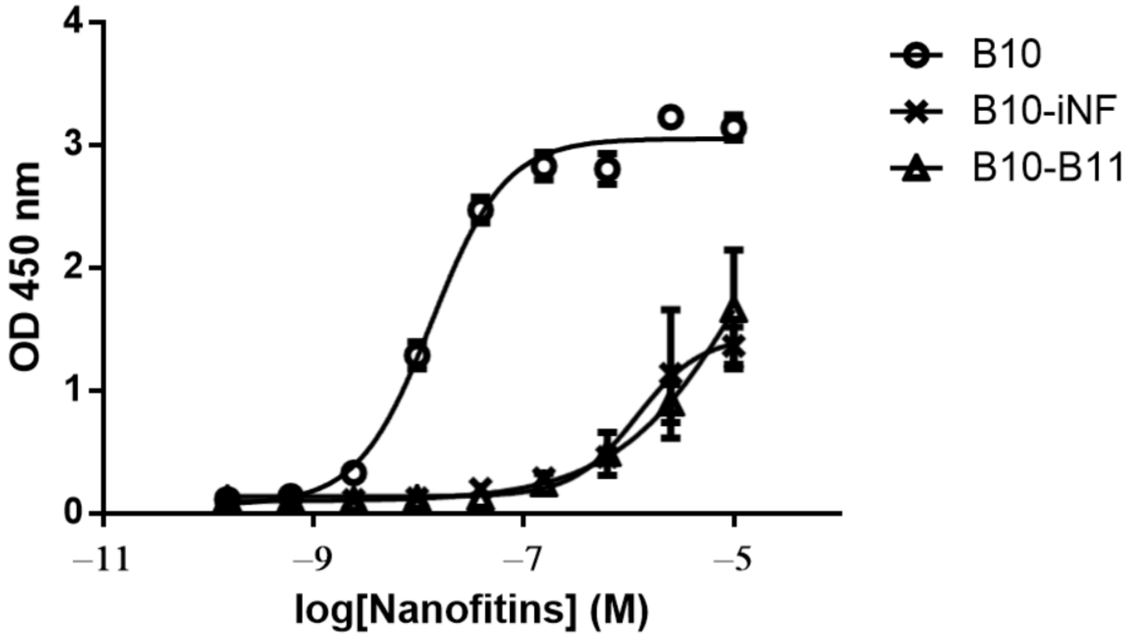


**Supplementary figure S2:** ELISA dose response curve on EGFR of the Nanofitin constructs B10, B10-iNF and B10-B11

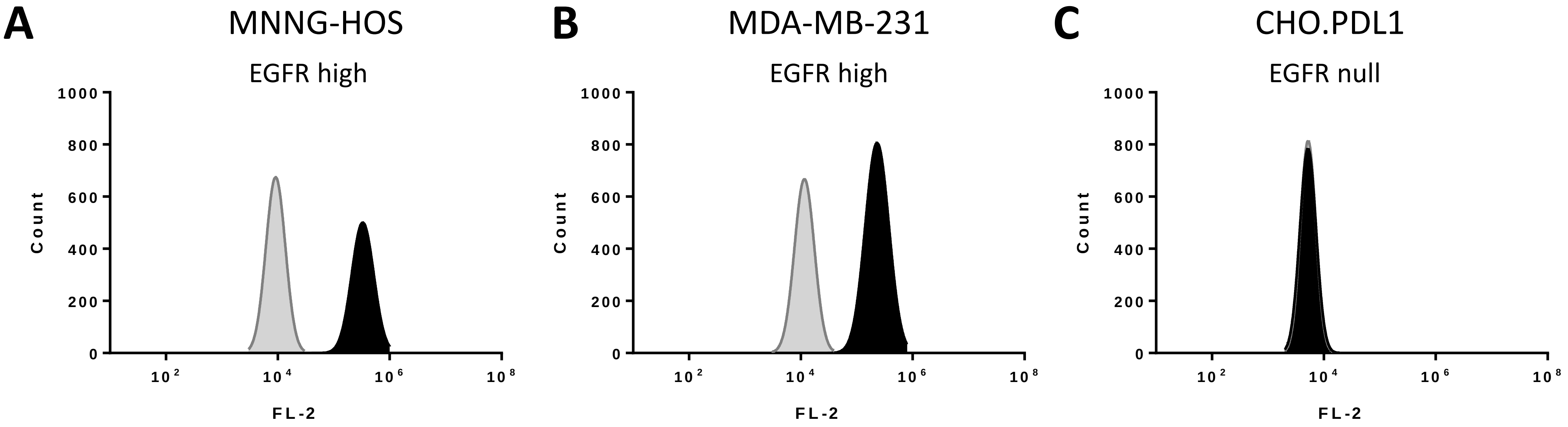


**<u>Supplementary figure S3:</u>** Expression level of EGFR studied by flow cytometry on (A) MNNG-HOS, (B) MDA-MB-231 and (C) on PDL1 expressing CHO cell lines. In grey : isotype control ; In black : anti-EGFR antibody

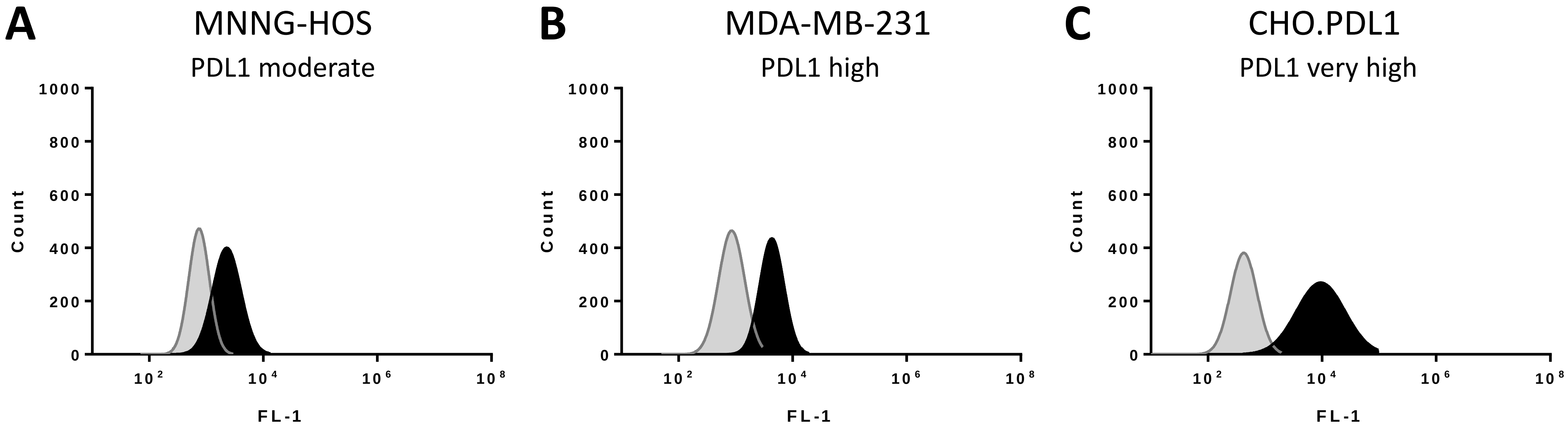


**Supplementary figure S4:** Expression level of PDL1 studied by flow cytometry on (A) MNNG-HOS, (B) MDA-MB-231 and (C) on PDL1 expressing CHO cell lines. In grey : isotype control ; In black : anti-PDL1 antibody

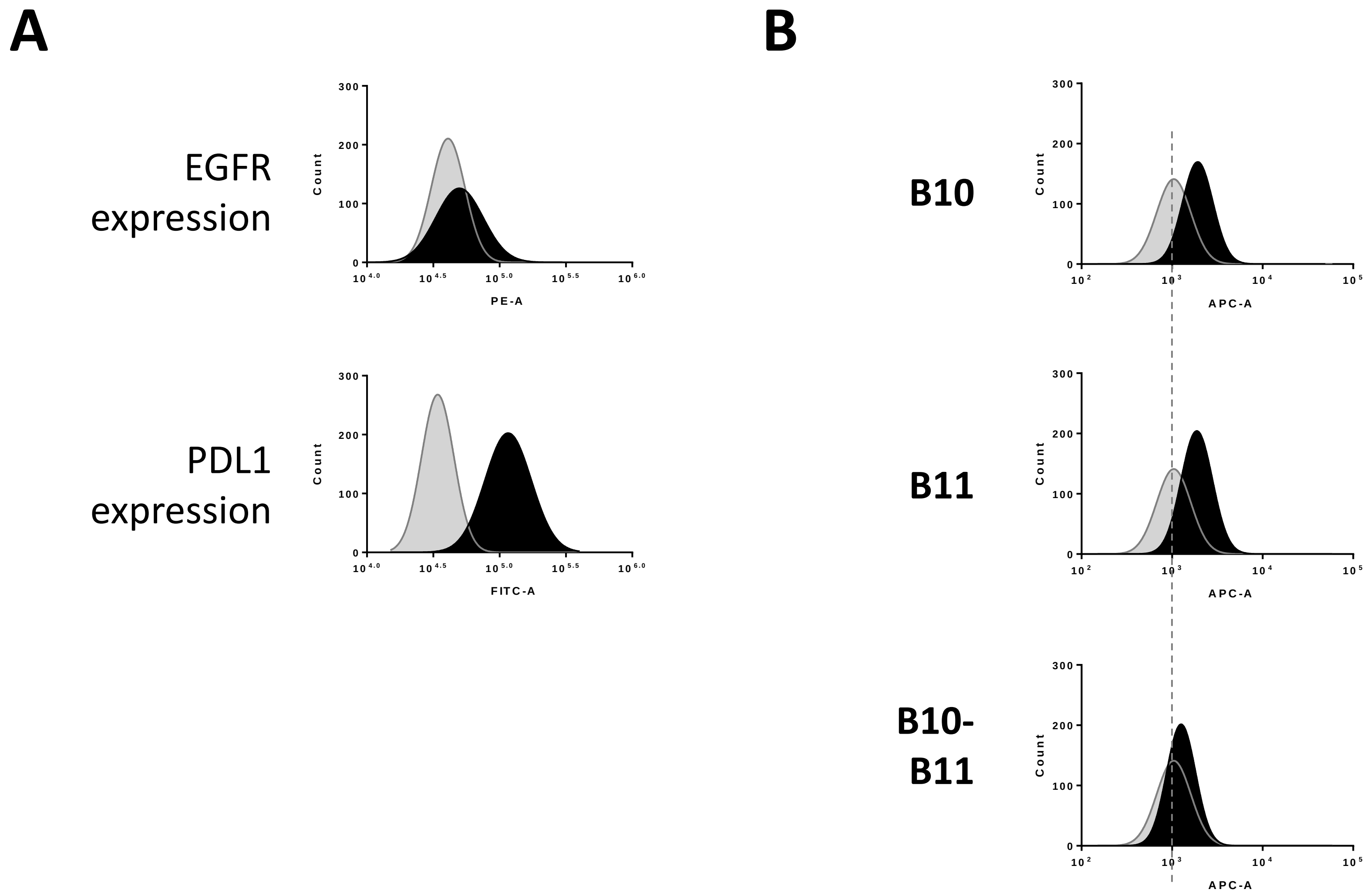


**Supplementary figure S5:** Flow cytometry labelling efficiency evaluation of Nanofitins on U2OS non target cell line (Low EGFR, High PDL1). (A) Expression level of EGFR and PDL1. In grey : isotype control ; In black : anti-EGFR or PDL1 antibody. (B) Cell labelling efficiency evaluation of B10, B11 and B10-B11 Nanofitins (10 µM). In grey : isotype control ; In black : the Nanofitin and secondary antibody.

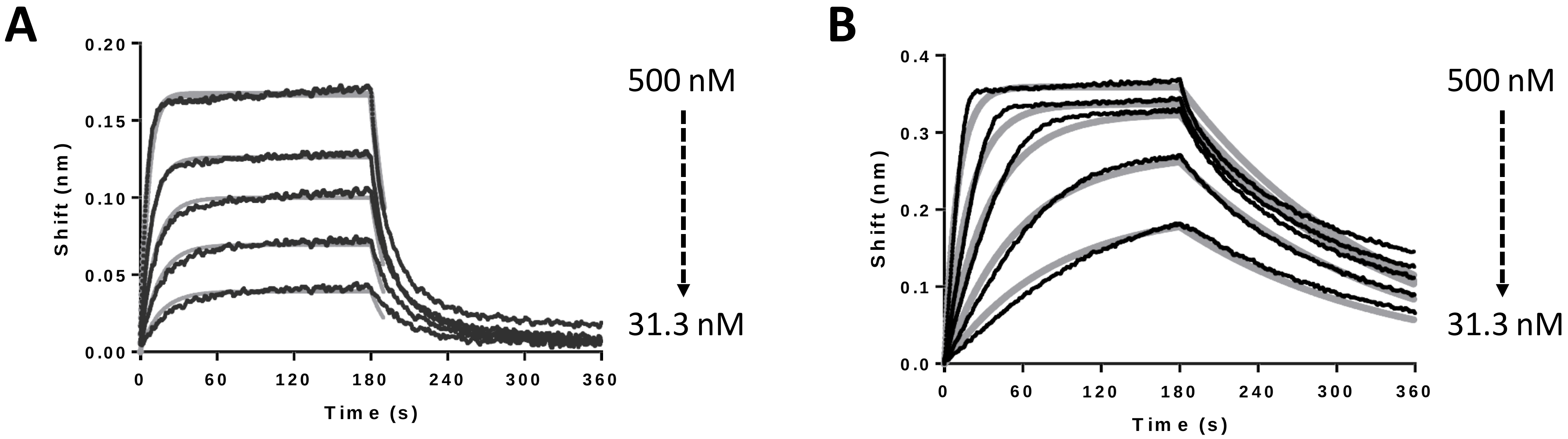


| KD (nM) | ka (1/Ms) | kdis (1/s) | $R^2$ |
|---|---|---|---|
| 245 | 2.41E+05 | 5.90E-02 | 0.9964 |

| KD (nM) | ka (1/Ms) | kdis (1/s) | $R^2$ |
|---|---|---|---|
| 34.6 | 1.84E+05 | 6.36E-03 | 0.9852 |

**Supplementary figure S6:** Affinity of the bispecific Nanofitin B10-B11 (5 mers linker) for (A) EGFR and (B) PDL1.

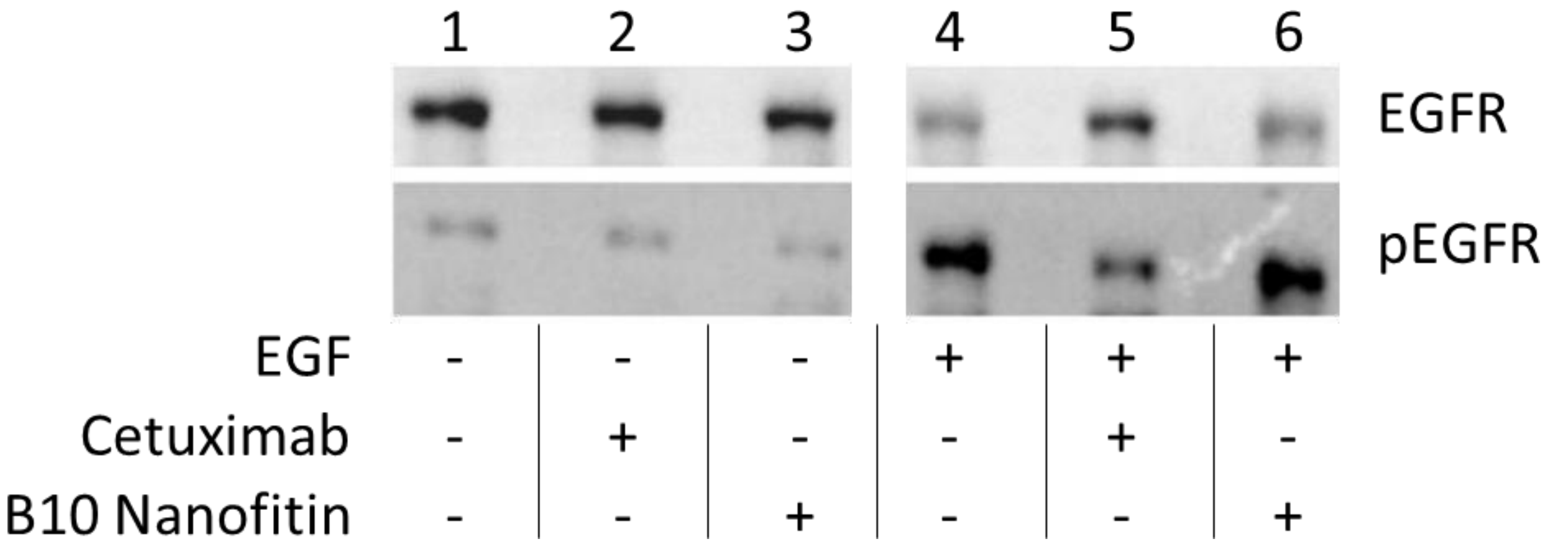


**Supplementary figure S7:** EGFR phosphorylation level in the presence or the absence of EGF, Cetuximab and B10 Nanofitin studied by Western Blot on A431 cell line.
