# Supplementary material for: Engineering of a Bispecific Nanofitin with Immune Checkpoint Inhibitory Activity Conditioned by the Cross-Arm Binding to EGFR and PDL1
Source: Biomolecules. 2023 Mar 31;13(4):636. doi: 10.3390/biom13040636 (PMC10135760; doi:10.3390/biom13040636)
Supplement: Supplementary file 1 [file biomolecules-13-00636-s001.zip › Supporting materials and methods.pdf]

SUPPORTING MATERIALS AND METHODS

1. Enzyme linked Immunosorbent Assays

EC50 of the Nanofitin constructs were measured by ELISA. 96 multi-well plates (VWR, #735-0083) were coated with recombinant human EGFR Fc chimera protein (R&D, #344-ER-050) diluted at 2.5 µg/mL in TBS1X buffer (100 µL/well, 1 hour,), washed with TBS1X buffer (300 µL/well) and blocked with TBS1X buffer containing 0.5% Bovine Serum Albumin (BSA) (300 µL/well, 1 hour) and then washed with TBS1X buffer containing 0.1% Tween 20 (300 µL/well). Serial dilutions of Nanofitins in TBS1X buffer containing 0.1% Tween 20 (TBS-T) were added (100 µL/well, 1 hour). After washing the plate with TBS-T (300 µL/well), an anti-RGS-His HRP conjugate antibody (Qiagen, #34450) diluted in TBS-T was added (100 µL/well, 1 hour). The plate was washed with TBS-T, and the revelation was performed by the addition of the TMB substrates (100 µL) then stopped by adding HCl (100 µL, 1M). All incubation steps were carried out at room temperature and 600 RPM and all plates were read at 450 nm (Varioskan system, Thermo Fisher Scientific, #3001-2017).

2. Biolayer interferometry analyses

Binding specificity on EGFR and PDL1 of the Nanofitins was determined by biolayer interferometry (BLI) on an Octet Red instrument (Fortebio). All biolayer interferometry analyses were performed in 96 multi-well plates (Dutsher, #655900) at 30°C with a continuous shake speed of 1000 RPM. The binding specificity of anti-EGFR Nanofitins was determined by loading of recombinant human EGFR Fc chimera protein (R&D, #344-ER-050) (10 µg/mL) at 2 nm on protein A biosensors (Sartorius, #18-5012). Similarly, the binding specificity of anti-PDL1 Nanofitins was determined by the loading of recombinant human PDL1/B7-H1 Fc chimera protein (R&D, #156-B7-100) (5 µg/mL) at 2 nm on protein A biosensors. All measured steps were performed in TBS1X containing 0.002% Tween 20 and 0.01% BSA. Between each measure, biosensors were regenerated using three cycles of alternating washes for 10 seconds in Glycine 10 mM pH 2 and in TBS1X. The biosensor unexposed to Nanofitin was used as a background reference. Sensorgrams were obtained after a reference subtraction, a background correction, a smoothing with the Savitzky-Golay algorithm and a fitting with a 1:1 model using the Octet Data Analysis software 7.1.

The binding kinetic parameters of anti-EGFR Nanofitins were determined by loading of recombinant human EGFR Fc chimera protein (R&D, #344-ER-050) (10 µg/mL) at 2 nm on protein A biosensors (Sartorius, #18-5012). Similarly, the binding kinetic parameters of anti-PDL1 Nanofitins were determined by the loading of recombinant human PDL1/B7-H1 Fc chimera protein (R&D, #156-B7-100) (5 µg/mL) at 2 nm on protein A biosensors. All measured steps were performed in TBS1X containing 0.002% Tween 20 and 0.01% BSA. Between each measure, biosensors were regenerated using three cycles of alternating washes for 10 seconds in Glycine 10 mM pH 2 and in TBS1X. The biosensor unexposed to Nanofitin was used as a background reference. Sensorgrams were obtained after a reference subtraction, a background correction, a smoothing with the Savitzky-Golay algorithm and a fitting with a 1:1 model using the Octet Data Analysis software 7.1.

3. Cell-surface binding by flow cytometry

U2OS proliferating cells were washed with PBS and detached by Accutase. After a 5 minutes centrifugation at 450x *g*, cells pellets were washed twice with cold PBS and then resuspended at $2x10^6$ cells/mL. Cells were distributed in 96 multi-well plates and incubated for 15 minutes in PBS1X-1%BSA. The expression level of EGFR and PDL1 on cells were studied by incubating cells with 100 µL of recombinant Alexa Fluor 488 anti-PD-L1 antibody (Abcam, #ab209959, 1/50 dilution) and PE anti-human EGFR antibody (Biolegend, #352903, 1/20 dilution) respectively. The isotype antibodies used were: Alexa Fluor 488 Rabbit IgG, monoclonal [EPR25A] (Abcam, #ab199091), PE mouse IgG1 kappa antibody (Biolegend, #400111). The cell binding capacity of Nanofitins was analyzed by incubating cells with 100 µL of Nanofitins at 10 µM, 1 µM and 0.1 µM followed by the addition of DyLight650 anti-HA tag antibody (Abcam, #ab117515, 1/200 dilution). To evaluate the Nanofitin cell-binding capacity, a control condition was performed by adding the DyLight650 anti-HA tag antibody on cells without previous incubation of Nanofitins. The FITC-A (Alexa Fluor 488), PE-A (PE) and APC-A (DyLight 650) fluorescence on cells were analyzed by flow cytometry (CytoFLEX – Beckman Coulter, Inc).
